# Prevalence and factors associated with maternal and neonatal sepsis in sub-Saharan Africa: a systematic review and meta-analysis

**DOI:** 10.3389/fpubh.2024.1272193

**Published:** 2024-01-24

**Authors:** Fatoumata Bintou Traoré, Cheick Sidya Sidibé, El Hadj Marouf Diallo, Bienvenu Salim Camara, Sidikiba Sidibé, Alhassane Diallo, Nielé Hawa Diarra, Birama Apho Ly, Mohamed Ali Ag Ahmed, Kassoum Kayentao, Abdoulaye Touré, Alioune Camara, Alexandre Delamou, Hamadoun Sangho, Ibrahim Terera

**Affiliations:** ^1^National Institute of Public Health, Bamako, Mali; ^2^African Center of Excellence for the Prevention and Control of Communicable Diseases, Gamal Abdel Nasser University of Conakry, Conakry, Guinea; ^3^Athena Institute for Research on Innovation and Communication in Health and Life Sciences, VU Amsterdam, Amsterdam, Netherlands; ^4^Department of Public Health, Gamal Abdel Nasser University of Conakry, Conakry, Guinea; ^5^Université de Montpellier, Montpellier, Languedoc-Roussillon, France; ^6^Faculté de Médecine et d'Odontostomatologie, Université des Sciences, des Techniques et des Technologies de Bamako, Bamako, Mali; ^7^Faculté de Pharmacie, Université des Sciences, Techniques et Technologies de Bamako, Bamako, Mali; ^8^Malaria Research and Training Center, Mali International Center for Excellence in Research, University of Sciences, Techniques, and Technologies of Bamako, Bamako, Mali; ^9^Center of Research and Training in Infectious Diseases, Gamal Abdel Nasser University of Conakry, Conakry, Guinea; ^10^National Malaria Control Programme Conakry, Conakry, Guinea

**Keywords:** maternal sepsis, neonatal sepsis, prevalence, associated factors, sub-Saharan Africa

## Abstract

**Objectives:**

This study aimed to determine the prevalence and factors associated with maternal and neonatal sepsis in sub-Saharan Africa.

**Methods:**

This systematic review and meta-analysis used the PRISMA guideline on sepsis data in sub-Saharan Africa. The bibliographic search was carried out on the following databases: Medline/PubMed, Cochrane Library, African Index Medicus, and Google Scholar. Additionally, the reference lists of the included studies were screened for potentially relevant studies. The last search was conducted on 15 October 2022. The Joanna Briggs Institute quality assessment checklist was applied for critical appraisal. Estimates of the prevalence of maternal and neonatal sepsis were pooled using a random-effects meta-analysis model. Heterogeneity between studies was estimated using the Q statistic and the I2 statistic. The funnel plot and Egger’s regression test were used to assess the publication bias.

**Results:**

A total of 39 studies were included in our review: 32 studies on neonatal sepsis and 7 studies on maternal sepsis. The overall pooled prevalence of maternal and neonatal sepsis in Sub-Saharan Africa was 19.21% (95% CI, 11.46–26.97) and 36.02% (CI: 26.68–45.36), respectively. The meta-analyses revealed that Apgar score < 7 (OR: 2.4, 95% CI: 1.6–3.5), meconium in the amniotic fluid (OR: 2.9, 95% CI: 1.8–4.5), prolonged rupture of membranes >12 h (OR: 2.8, 95% CI: 1.9–4.1), male sex (OR: 1.2, 95% CI: 1.1–1.4), intrapartum fever (OR: 2.4, 95% CI: 1.5–3.7), and history of urinary tract infection in the mother (OR: 2.7, 95% CI: 1.4–5.2) are factors associated with neonatal sepsis. Rural residence (OR: 2.3, 95% CI: 1.01–10.9), parity (OR: 0.5, 95% CI: 0.3–0.7), prolonged labor (OR: 3.4, 95% CI: 1.6–6.9), and multiple digital vaginal examinations (OR: 4.4, 95% CI: 1.3–14.3) were significantly associated with maternal sepsis.

**Conclusion:**

The prevalence of maternal and neonatal sepsis was high in sub-Saharan Africa. Multiple factors associated with neonatal and maternal sepsis were identified. These factors could help in the prevention and development of strategies to combat maternal and neonatal sepsis. Given the high risk of bias and high heterogeneity, further high-quality research is needed in the sub-Saharan African context, including a meta-analysis of individual data.

**Systematic review registration:** PROSPERO (ID: CRD42022382050).

## Introduction

Maternal and neonatal sepsis is a generalized inflammatory response with systemic manifestations caused by one or more infectious agents (1), which occurs during pregnancy, childbirth, after abortion, or during the postpartum period (42 days) in women or in the first 28 days of life in newborns (2–4). Sepsis is a major cause of maternal and neonatal morbidity and mortality (2, 3, 5–9). It is the third leading cause of death in women and accounts for a quarter of neonatal deaths (1, 10–14). It is an obstacle to achieving the third Sustainable Development Goal (SDG), which aims to reduce maternal and neonatal mortality and morbidity (15). Low- and middle-income countries (LICs) are particularly affected by maternal and neonatal sepsis (11, 16, 17). In sub-Saharan Africa (SSA), it is estimated to be responsible for 130,000 maternal deaths and 300,000 neonatal deaths per year, although this may be an underestimation (2, 18). These deaths reflect a number of challenges, including policy, poverty, health inequalities, and the health system (19). However, few data are available on the prevalence and factors associated with sepsis across the continuum from pregnancy to postpartum or post-abortion, making it difficult to make a real estimation of maternal and neonatal sepsis in these countries (2, 10, 11). In the literature, the factors associated with maternal and neonatal sepsis are diverse, including prolonged labor, failure to perform antenatal consultation (ANC), prolonged rupture of membranes, history of infection in the mother, repeated vaginal examinations, intrapartum fever, gestational age, parity, type of delivery, prematurity, chorioamnionitis, meconium-stained amniotic fluid, Apgar score < 7, low birth weight < 2.5 kg, resuscitation of the newborn, age of the newborn <7 days, and male sex (5–7, 20–23).

Prevention, early recognition of signs, and rapid and appropriate management of cases are the main factors associated with a reduction in the morbidity and mortality associated with maternal and neonatal sepsis (5, 11, 24–26). A recent meta-analysis carried out in SSA in 2022 identified several risk factors for neonatal sepsis (27) but did not explore the magnitude of neonatal and/or maternal sepsis nor the factors associated with maternal sepsis. Other studies have shown that the prevalence of maternal sepsis was 39% in Ethiopia (28), 12.20% in Keyna (29), and 20% in Tanzania (30); for neonatal sepsis, it was 77.9 in Ethiopia (31), 20.5% in South Africa (32), 49.8 in Tanzania (33), 37.6 in Nigeria (34), and 17.5 in Ghana (35). Although these single studies reported data on the prevalence and factors associated with maternal and neonatal sepsis, there are no regionally representative pooled data on the magnitude and factors associated with maternal and neonatal sepsis in SSA. However, a better understanding of the burden and a synthesis of the evidence on the factors associated with maternal and neonatal sepsis are needed to optimize prevention strategies and management guidelines against this scourge. The aim of this systematic review with meta-analysis was to estimate the prevalence and factors associated with maternal and neonatal sepsis in sub-Saharan Africa. To the best of our knowledge, this is the first systematic review and meta-analysis to examine both the prevalence of and factors associated with maternal and neonatal sepsis in SSA. It aimed to answer the following questions:

What is the prevalence of maternal and neonatal sepsis in sub-Saharan Africa?
What are the associated factors with maternal and neonatal sepsis in sub-Saharan Africa?

## Materials and methods

This systematic review was reported in accordance with PRISMA (Preferred Reporting Item for Systematic Review and Meta-analysis) guidelines (36). The protocol for this review was developed and registered in the “International prospective register of systematic reviews PROSPERO” (ID: CRD42022382050).

### Search strategies

To identify eligible studies, Medline/PubMed, Cochrane Library, African Index Medicus, and Google Scholar databases were searched. We also conducted manual searches of the bibliographic references of included studies and meta-analyses. The adapted PECO format was used for this systematic review. This PECO included population (P), exposure (E), comparison (C), and outcome (O), as shown in Table 1. It consisted of using all the identified keywords and indexing terms to search different databases. All the search terms used and the MeSH terms for the search were added, as well as the Boolean operators, to guarantee the exhaustiveness of the search process. Key terms defining the same concept were introduced using the “OR” operator, and the “AND” operator was used to introduce different concepts.

**Table 1:** PECOT framework for the review objective.

Components	Characteristics
Population	Pregnant women, women in labor, postpartum women, newborns,
Exposure	Associated factors: prolonged labor, failure to perform ANC, prolonged rupture of membranes, history of infection in the mother, repeated vaginal examinations, intrapartum fever, gestational age, parity, cesarean delivery, prematurity, meconium amniotic fluid, Apgar score < 7, birth weight < 2.5 kg, age of newborn <7 days, prematurity.
Comparison	Absence of exposure
Results	Maternal sepsis and associated factors

### Selection of studies/eligibility criteria

The result of bibliographic searches carried out on the various search tools was exported to Zotero, where duplicates were identified and removed using the Duplicates command and manually also removed during the screening. The study selection process followed two evaluation stages (37). The first evaluation was based on an examination of the titles and abstracts of the articles. The titles and abstracts that were outside the scope of the study were excluded. The second assessment consisted of examining the full text of eligible studies. The entire process was carried out by two reviewers (FBT and NHD). They independently performed abstract screening and full-text study selection, where both authors had to approve the inclusion of the study in the systematic review. The reference lists of the included studies were screened for potentially relevant studies (38).

Studies reporting on the prevalence and/or at least one factor associated with maternal and/or neonatal sepsis in SSA in pregnant women from 28 weeks of amenorrhea (SA), postpartum women up to 42 days after delivery, post-abortion women, and newborns within 28 days of birth were included. Cross-sectional, cohort, and case–control studies on the prevalence, frequency, and factors associated with maternal and neonatal sepsis in SSA published in French and English between January 2012 and October 2022 were included. Qualitative studies, systematic reviews, and case series were excluded from the analysis, but the reference lists of these were screened.

### Assessment of study quality and risk of bias

The quality of the included studies was assessed by two authors (FBT and NHD) using the Joanna Briggs Institute (JBI) quality assessment checklist (39). For cross-sectional studies, the following criteria were used: (1) conformity between target population and source population; (2) appropriate sampling technique; (3) representativeness of the sample; (4) description of the subject and context of the study; (5) data analysis with sufficient sample coverage; (6) valid methods for identifying the condition; (7) standard and reliable way of measuring the condition for all participants; (8) appropriate statistical test; and (9) adequate response rate. When items received a score ≥ 6 out of 9, they were considered to be of high quality. The following were used to assess cohort studies: (1) similarity of groups, (2) similarity of exposure measurement, (3) validity and reliability of measurement, (4) identification of confounders, (5) strategies for dealing with confounders, (6) adequacy of groups/participants at study entry, (7) validity and reliability of measured outcomes, (8) sufficient duration of follow-up, (9) completeness of follow-up or description of reasons for loss to follow-up, (10) strategies for dealing with incomplete follow-up, and (11) adequacy of statistical analysis. The criteria used to evaluate case–control studies are (1) comparable groups, (2) appropriateness of cases and controls, (3) criteria for identifying cases and controls, (4) standard exposure measurement, (5) similarity of exposure measurement for cases and controls, (6) treatment of confounding factors, (7) strategies for treating confounding factors, (8) standard evaluation of results, (9) appropriateness of duration of exposure, and (10) appropriateness of statistical analysis (see Table 2).

**Table 2:** Quality assessment results of included studies in sub-Saharan Africa from January 2002–October 2022 using the Joanna Briggs Institute (JBI) quality appraisal checklist.

Author	Quality assessment questions												
	Q1	Q2	Q3	Q4	Q5	Q6	Q7	Q8	Q9	Q10	Q11	Yes Total	Quality status
**Cross-sectional studies**													
Tsehaynesh G/eyesus	N	Y	Y	UC	Y	Y	UC	Y	UC			5/9	Medium risk
Kumera Bekele	Y	Y	Y	UC	Y	Y	Y	Y	UC			7/9	Low risk
Abebe Sorsa	Y	UC	Y	Y	UC	Y	Y	UC	Y			6/9	Low risk
Abimbola Ellen Akindolire	Y	Y	Y	UC	Y	Y	Y	Y	Y			8/9	Low risk
Fortress Yayra Aku	UC	UC	Y	N	Y	Y	Y	Y	UC			5/9	Medium risk
Alemnew Wale	UC	Y	Y	UC	Y	UC	Y	Y	N			5/9	Medium risk
Bua John, 2015	N	Y	N	Y	Y	UC	Y	Y	Y			6/9	Low risk
Debora C. Kajegukaa, 2020	UC	Y	Y	N	UC	Y	Y	Y	UC			5/9	Medium risk
Aytenew Getabelew, 2018	Y	Y	Y	N	N	N	Y	Y	UC			5/9	Medium risk
Tchouambou SN Clotilde, 2022	UC	N	Y	UC	N	Y	Y	Y	Y			5/9	Medium risk
Abdulhakeem Abayomi Olorukooba, 2020	Y	Y	Y	Y	Y	N	UC	Y	Y			7/9	Low risk
Mekitrida L. Kiwone, 2020	UC	N	Y	Y	UC	Y	Y	Y	UC			5/9	Medium risk
Tilahun Tewabe, 2017	Y	Y	Y	UC	Y	Y	Y	UC	Y			7/9	Low risk
Endalk Birrie, 2020	Y	Y	Y	Y	Y	Y	Y	Y	Y			9/9	Low risk
Abdurahman Kedir Roble, 2022	UC	Y	Y	Y	UC	Y	Y	Y	UC			7/9	Low risk
Zelalem Agnche, 2020	Y	Y	Y	Y	Y	UC	Y	Y	Y			8/9	Low risk
Daniel Atlaw, 2019	Y	Y	Y	UC	Y	Y	Y	Y	Y			8/9	Low risk
Alemale Admas, 2020	Y	Y	Y	U	UV	Y	Y	Y	Y			8/9	Low risk
Yenew Engida Yismaw, 2019	Y	Y	Y	UC	Y	UC	Y	UC	Y			6/9	Low risk
Tinuade A Ogunlesi, 2010	Y	Y	Y	Y	UC	Y	Y	Y	Y			8/9	Low risk
Agricola Joachim, 2009	Y	Y	Y	UC	Y	Y	Y	Y	Y			8/9	Low risk
Neema Kayange, 2010	Y	Y	Y	UC	Y	UC	UC	Y	Y			6/9	Low risk
Ogundare Ezra Olatunde, 2015	Y	Y	Y	UC	Y	Y	Y	Y	Y			8/9	Low risk
BA West, 2014	Y	Y	UC	Y	UC	N	Y	Y	Y			6/9	Low risk
**Cohort studies**													
Violet Okaba Kayom	Y	Y	N	Y	UC	Y	Y	Y	Y	N	Y	8/11	Low risk
Shatry N. A., 2022	Y	Y	Y	Y	UC	Y	Y	N	UC	Y	Y	8/11	Low risk
**Case–control study**													
Mulunesh Alemu	Y	Y	Y	UC	Y	Y	Y	Y	UC	Y		8/10	Low risk
Kalkidan Béjituel	Y	Y	Y	UC	Y	Y	Y	Y	Y	Y		9/10	Low risk
Getu Alemu Demisse	Y	Y	Y	UC	Y	Y	Y	Y	N	Y		8/10	Low risk
Dejene Edosa Dirirsa	Y	Y	Y	UC	Y	Y	UC	Y	Y	Y		8/10	Low risk
Gujo Teshome	Y	Y	Y	Y	Y	Y	N	Y	UN	Y		8/10	Low risk
Peter Adatara, 2019	Y	Y	Y	UC	Y	N	UC	Y	Y	Y		7/10	Medium risk
Peter Adatara, 2018	Y	Y	Y	N	UN	Y	Y	UC	UC	Y		6/10	Medium risk
Destaalem Gebremedhin	Y	Y	Y	Y	Y	UC	UC	Y	Y	Y		8/10	Low risk
Pendo P. Masanja	Y	Y	Y	Y	Y	N	N	Y	UC	Y		7/10	Low risk
Atkuregn Alemayehu, 2020	Y	N	Y	Y	Y	UC	UC	Y	Y	Y		7/10	Low risk
Tadesse Yirga AkaluID, 2020	Y	Y	Y	U	UC	UC	Y	Y	Y	Y		8/10	Low risk
Soressa Gemechu Kitessa, 2021	Y	Y	Y	Y	Y	UC	UC	Y	Y	Y		8/10	Low risk
Mate Siakwa, 2014	Y	Y	Y	UC	UC	N	UC	Y	Y	Y		6/10	Medium risk

Q, Question; Y, yes; N, no; UC, unclear.

### Data extraction

Two authors (FBT and NHD) independently extracted data using a tested form on Microsoft Excel. If discrepancies between data extractors continued, a third reviewer (EMD) was involved. Data extracted included author name and year of publication, country, study period, study setting, study design, study population, sample size, type of sepsis (maternal or neonatal), prevalence of neonatal sepsis, prevalence of maternal sepsis, and risk bias.

For the associated factors, data were extracted on the age of the newborn, Apgar score, gestational age, birth weight, resuscitation of the newborn at birth, sex of the newborn, antenatal consultation (ANC), presence of prolonged rupture of membranes, repeated vaginal examinations, intrapartum fever, gestational age, parity, type of delivery, prematurity, meconium amniotic fluid, history of infection in the mother, maternal fever, type of delivery, and prolonged labor.

### Statistical analysis

Data were entered into Microsoft Excel and then exported into Stata version 17 software. We pooled maternal and neonatal sepsis prevalence estimates using a random-effects meta-analysis model because it accounts for variability between studies. We examined the heterogeneity of effect size using the Q statistic and the I^2^ statistic (40). An I^2^ value ≥ 50% was considered strong heterogeneity, and a random-effects model was used (41, 42). The random-effect RELM method was mainly used. The funnel plot and Eggers’s regression test were used to check for publication bias (43). To complete the tests for publication bias, we added the Begg and Thompson tests on R software. Forest plots were used to display the results graphically. A subgroup analysis was performed according to study design (prospective or retrospective), sepsis diagnostic criteria (clinic or clinic and biology), risk of bias (low or moderate), and different regions of SSA (East, West, or South Africa).

## Results

**Figure 1:**
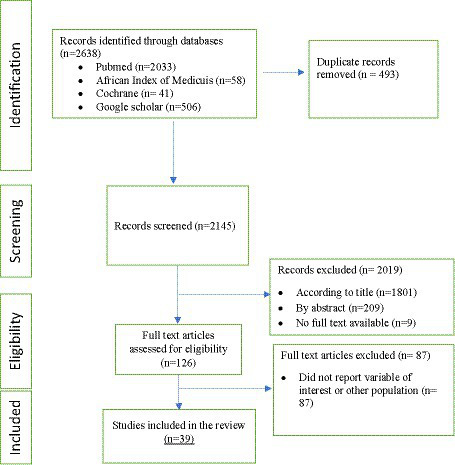
PRISMA flow diagram describing the results of study selection.

A total of 2,638 titles were screened (2,033 from PubMed, 58 from the African Index of Medicus, 41 from Cochrane, and 506 from Google Scholar). After removing duplicates, 2,145 studies remained. Evaluation of titles and abstracts led to the exclusion of 2,019 studies. Nine studies were excluded because the full text was not available. The full-text review involved 126 studies. The number of articles retained for inclusion was 39. The reasons for the exclusion of 87 studies were the lack of availability of the variable of interest and the difference in the target population (Figure 1).

### Characteristics of the studies included in the meta-analysis

A total of 39 studies were included in our review: 32 studies of neonatal sepsis and 7 studies of maternal sepsis. There were 24 cross-sectional studies (22, 30–34, 44–60), 13 case–control studies (28, 35, 61–69), and 2 cohorts (29, 70).

Twenty-one studies were from Ethiopia (28, 31, 44–46, 49, 51–56, 60–64, 66, 68, 69, 71), five from Tanzania (22, 30, 33, 67, 72), five from Nigeria (34, 47, 59, 73, 74), four from Ghana (35, 48, 65, 75), two from Uganda (50, 70), one from Kenya (29), and one from South Africa (32). The total study population was 12,777, including 10,494 newborns and 2,283 women. After quality assessment, 30 studies had a low risk of bias and 9 had a medium risk of bias (see Table 3).

**Table 3:** Characteristics of included studies.

*N*	First author, publication year	Country	Study period	Setting	Study design	Study population	Sample size	Type of sepsis	Prevalence	Risk of bias
1	Tsehaynesh G/eyesus, 2017	Ethiopia	September 2015 to May 2016	Hospital	Prospective Cross-sectional	Newborn	251	Neonatal	46.61%	Medium risk
2	Kumera Bekele, 2022	Ethiopia	January 2021 to March 2021	Hospitals	Transversale prospective	Newborn	378	Neonatal	52.27%.	Low risk
3	Mulunesh Alemu, 2019	Ethiopia	1 February to 30 March 2018	Hospitals	Case-control	Newborn	246	Neonatal	-	Low risk
4	Kalkidan Béjituel, 2022	Ethiopia	1 August to 30 September 2020	Hospitals	Case-control	Newborn	331	Neonatal	-	Low risk
5	Getu Alemu Demisse, 2019	Ethiopia	1 February to 30 April 2018	Hospitals	Case-control	Mother	280	Maternal	-	Low risk
6	Dejene Edosa Dirirsa, 2021	Ethiopia	May 2018 to August 2018	Hospitals	Case-control	Newborn	220	Neonatal		Low risk
7	Abebe Sorsa, 2019	Ethiopia	April 2016 to May 2017	Hospital	Prospective Cross-sectional	Newborn	303	Neonatal	34%	Low risk
8	Abimbola Ellen Akindolire, 2016	Nigeria	November 2013 and February 2014	Hospitals	Prospective Cross-sectional	Newborn	202	Neonatal	12.37	Low risk
9	Fortress Yayra Aku, 2020	Ghana	January and May 2016	Hospitals	Prospective Cross-sectional	Newborn	150	Neonatal	17.3%	Medium risk
10	Alemnew Wale, 2021	Ethiopia	May to November 2019	Hospital	Prospective Cross-sectional	Newborn	193	Neonatal	26.1%	Medium risk
11	Gujo Teshome, 2022	Ethiopia	1 October to 10 November 2021	Hospitals	Case-control	Newborn	293	Néonatal	-	Low risk
12	Peter Adatara, 2019	Ghana	January and December 2017	Hospital	Case-control	Newborn	900	Neonatal	-	Low risk
13	Peter Adatara, 2018	Ghana	4 weeks	Hospital	Case-control	Newborn	383	Neonatal	17.50%	Medium risk
14	Destaalem Gebremedhin, 2016	Ethiopia	December 2014 to June 2015	Hospitals	Case-control	Newborn	234	Neonatal	-	Low risk
15	Bua John, 2015	Uganda	January and August 2013	Health Center	Prospective Cross-sectional	Mother and newborn	174	Neonatal	21.80%	Low risk
16	Debora C. Kajegukaa, 2020	Tanzania	January 2015 to December 2015	Hospital	Retrospective Cross-sectional	Mother	183	Maternal	11.5%	Medium risk
17	Violet Okaba Kayom, 2018	Uganda	March to May 2012	Community	Prospective Cohort	Mother and newborn	335	Neonatal		Low risk
18	Pendo P. Masanja, 2019	Tanzania	May to July 2017	Hospitals	Case–control	Mother and newborn	322	Neonatal		Low risk
19	Atkuregn Alemayehu, 2020	Ethiopia	April to July 2019	Hospitals	Case–control	Newborn	385	Neonatal		Low risk
20	Tadesse Yirga AkaluID, 2020	Ethiopia	March 2018 to April 2018	Hospitals	Case–control	Newborn	231	Neonatal		Low risk
21	Aytenew Getabelew, 2018	Ethiopia	1 February 2016 to 1 February 2017	Hospitals	Retrospective Cross-sectional	Newborn	224	Neonatal	77.9%	Medium risk
22	Tchouambou SN Clotilde, 2022	South Africa	1 January and 30 June 2018	Hospitals	Prospective Cross-sectional	Newborn	210	Neonatal	20.5%	Medium risk
23	Abdulhakeem Abayomi Olorukooba, 2020	Nigeria	May 2017 to May 2018	Hospital	Retrospective Cross-sectional	Newborn	409	Neonatal	37.6%.	Low risk
24	Mekitrida L. Kiwone, 2020	Tanzania	August to October 2018	Hospital	Retrospective Cross-sectional	Newborn	263	Neonatal	49.8%	Medium risk
25	Tilahun Tewabe, 2017	Ethiopia	30 April to 30 May 2016	Hospital	Retrospective Cross-sectional	Newborn	225	Neonatal		Low risk
26	Endalk Birrie, 2020	Ethiopia	1 January to 30 July 2021	Hospital	Prospective Cross-sectional	Newborn	344	Neonatal	79.4%	Low risk
27	Abdurahman Kedir Roble, 2022	Ethiopia	1 January 2019 to 31 December 2019	Hospitals	Prospective Cross-sectional	Newborn	361	Neonatal	45.80%	Low risk
28	Zelalem Agnche, 2020	Ethiopia	March to April 2019	Hospitals	Prospective Cross-sectional	Mother and newborn	352	Neonatal	64.8%	Low risk
29	Soressa Gemechu Kitessa, 2021	Ethiopia	May to October 2020	Hospitals	Case–control	Mother	428	Maternal	−39%	Low risk
30	Shatry N. A., 2022	Kenya	March to November 2015	Hospital	Prospective Cohort	Mother	566	Maternal	12.20%	Low risk
31	Daniel Atlaw, 2019	Ethiopia	1 September to 30 December 2017	Hospital	Prospective Cross-sectional	Mother	219	Maternal	17.2%,	Low risk
32	Alemale Admas, 2020	Ethiopia	January to May 2017	Hospital	Prospective Cross-sectional	Mother	166	Maternal	33.70%	Low risk
33	Ayenew Engida Yismaw, 2019	Ethiopia	1st September to 30th November 2017	Hospital	Prospective Cross-sectional	Newborn	423	Neonatal	11.70%	Low risk
34	Tinuade A Ogunlesi, 2010	Nigeria	January 2006 to December 2008	Hospital	Retrospective Cross-sectional	Newborn	1,050	Neonatal	16.5	Low risk
35	Agricola Joachim, 2009	Tanzania	October 2008 to March 2009	Hospital	Prospective Cross-sectional	Mother	300	Maternal	20%	Low risk
36	Neema Kayange, 2010	Tanzania	March to November 2009	Hospital	Prospective Cross-sectional	Newborn	770	Neonatal	39%	Low risk
37	Mate Siakwa, 2014	Ghana	January 2011 and December 2013	Hospital	Case-control	Newborn	196	Neonatal		Medium risk
38	Ogundare Ezra Olatunde, 2015	Nigeria	September 2008 to March 2009	Hospital	Prospective Cross-sectional	Newborn	360	Neonatal	16%	Low risk
39	BA West, 2012	Nigeria	July to December 2007	Hospital	Prospective Cross-sectional	Newborn	406	Neonatal	41.6%	Low risk

### Prevalence of maternal sepsis

**Figure 2:**
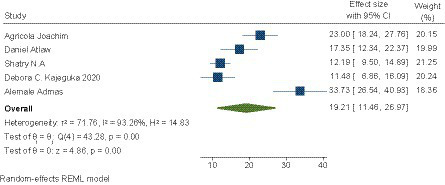
Pooled prevalence of maternal sepsis in SSA.

The pooled prevalence of maternal sepsis in SSA was 19.21% (95% CI, 11.46–26.97). Significant heterogeneity was observed between studies (I^2^ = 93.26%, *p* < 0.000). A random effects model was used to measure pooled prevalence. The highest prevalence was reported by Alemale et al. (54) (33.7%) and the lowest by Debora et al. (22) (11.5%); Figure 2 shows the details.

### Subgroup analysis of the prevalence of maternal sepsis

A subgroup analysis of the prevalence of maternal sepsis was carried out according to the diagnostic criteria for sepsis (clinical or clinical and biological). It was 25.33 (9.28–41.38) for sepsis based on clinical signs and 15.43 (8.28–22.58) for sepsis based on clinical signs and biology (22, 29, 30). See Table 4 for details.

**Table 4:** Sub-group analysis of the prevalence of maternal sepsis in sub-Saharan Africa.

Variable	Characteristics	Pooled prevalence (95% CI)	I^**2**^ (value of *p*)
Diagnostic criteria	Clinical	25.33 (9.28–41.38)	92.54% (<0.000)
	Clinical and biological	15.43 (8.28–22.58)	89.72% (<0.000)

### Prevalence of neonatal sepsis

**Figure 3:**
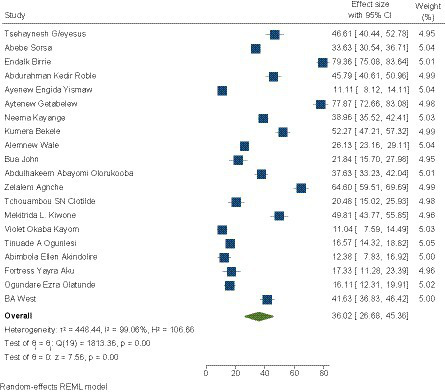
Pooled prevalence of neonatal sepsis in SSA.

The pooled prevalence of neonatal sepsis in SSA was 36.02% (95% CI, 26.68–49.36). A significant heterogeneity between the included studies was observed (I2 = 99.1%, *p* < 0.000). Therefore, a random effects model was used to estimate the pooled prevalence. The prevalence ranged from 11.1 (56) up to 77.9% (31) (see Figure 3).

### Subgroup analysis of the prevalence of neonatal sepsis

A subgroup analysis of prevalence was performed according to the design (prospective and retrospective), subdivision of SSA (East, West, or South Africa), definition of sepsis (clinical when diagnostic based on clinical signs or clinical and biological when diagnostic based on clinical signs and confirmed by biological tests), and risk of bias (low or moderate).

Depending on the design, the prevalence of neonatal sepsis was 32.88 (22.35–43.41) for prospective studies and 45.46 (25.97–64.94) for retrospective studies. According to the diagnostic criteria for sepsis, it was 44.11 (27.33–60.90) for sepsis based on clinical signs and 31.25 (22.24–40.26) for sepsis based on clinical signs and biology. According to the subdivision, the prevalence of neonatal sepsis was 42.96 (30.75–55.16) in East Africa, 23.59 (15.53–33.65) in West Africa, and 20.48 (15.0–25.93) in South Africa. The prevalence of neonatal sepsis was 34.45 (23.93–44.98) for studies with a low risk of bias and 39.69 (21.58–57.80) for studies with a medium risk of bias (see Table 5).

**Table 5:** Sub-group analysis of the prevalence of neonatal sepsis in sub-Saharan Africa.

Variables	Characteristics	Pooled prevalence (95% CI)	I^**2**^ (value of *p*)
Countries	East Africa	42.96 (30.75–55.16)	97.41% (<0.000)
	West Africa	23.59 (15.53–33.65)	98.98% (<0.000)
	South Africa	20.48 (15.0–25.93)	0%
Study design	Foresight	32.88 (22.35–43.41)	98.98% (<0.000)
	Retrospective	45.46 (25.97–64.94)	99.05% (<0.000)
Diagnostic criteria	Clinical	44.11 (27.33–60.90)	99.37% (<0.000)
	Clinical and biological	31.25 (22.24–40.26)	97.64% (<0.000)
Risk of bias	Low	34.45 (23.93–44.98)	99.06% (<0.000)
	Medium	39.69 (21.58–57.80)	98.95% (<0.000)

### Publication bias

**Figure 4:**
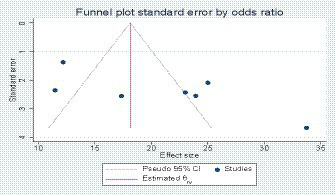
Funnel plot showing publication bias in maternal sepsis studies.

We assessed publication bias using the funnel plot and Egger’s regression test (76). Compared with the maternal sepsis studies, a visual asymmetrical distribution was observed, and Egger’s regression test (value of *p* = 0.01) indicated the presence of a publication bias (see Figure 4). We did not use the Begg and Thompson tests due to the number of studies less than 10.

**Figure 5:**
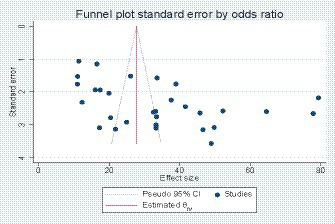
Funnel plot showing publication bias d in neonatal sepsis studies.

For the study concerning neonatal sepsis, we assessed publication bias using the funnel plot, Egger’s regression test, Begg’s test, and Thompson test. The diagrams showed asymmetry, and the result of Egger’s test showed the presence of bias (*p* = 0.04; see Figure 5). However, Begg’s test (value of *p* = 0.06) and the Thompson test (value of *p* = 0.14) did not reject the city Ho. Therefore, the asymmetry on the funnel plot is reflected much more by a small study effect than by a publication bias.

### Meta-analysis of factors associated with maternal sepsis

A total of eight risk factors were included in the meta-analysis: place of residence for 3 studies (22, 28, 63), parity for 3 studies (22, 30, 54), mode of delivery for 5 studies (22, 28, 54, 60, 63), prolonged labor for 5 studies (22, 28, 29, 54, 63), multiple vaginal examinations for 3 studies (28, 29, 63), performance of ANC for 4 studies (28, 54, 60, 63), history of urinary tract infection for 3 studies (22, 29, 30), and level of education for 4 studies (28, 29, 60, 63). Table 6 shows the details.

**Table 6:** Risk factors included in the meta-analysis for maternal sepsis.

Result	Comparison	Number studies	Effect size	Pooled estimate	Q	Heterogeneity I^**2**^, value of *p*
Residence	Rural, urban	3	Odds ratio	3.32 (1.1–10.96)	20.64	90%, 0.048
Parity	Primiparous, multiparous	3	Odds ratio	0.50 (0.32–0.78)	0.42	0.0%, 0.003
Delivery method	CS/ VD	5	Odds ratio	1.47 (0.54–4.01)	35.70	88.8%, 0.448
Prolonged labor	>12 h,<12 h	5	Odds ratio	3.37 (1.64–6.95)	21.60	81.5%, 0.001
Multiple vaginal examinations	>5, <5	3	Odds ratio	4.33 (1.31–14.36)	20.73	90.4%, 0.016
ANC	<4, ≥4	4	Odds ratio	1.849 (0.77–4.42)	15.65	80.8%, 0.168
History of urinary tract infections	Yes or No	3	Odds ratio	1.672 (0.71–3.90)	5.02	60.2%, 0.234
Level of education	No education, education	4	Odds ratio	1.32 (0.93–1.86)	0.99	0.0%, 0.112

CS, Cesarean section; VD, Vaginal delivery.

### Meta-analysis of factors associated with neonatal sepsis

A meta-analysis was carried out for 15 risk factors classified into maternal and neonatal factors. The maternal factors were history of urinary tract infection in the mother (13 studies) (31, 45, 53, 55, 56, 61, 62, 65, 66, 68–71), parity (10 studies) (31, 45, 48, 53, 56, 61, 62, 65, 67, 68), prolonged labor (5 studies) (31, 53, 62, 69, 73), intrapartum fever (13 studies) (31, 45, 48, 52, 55, 56, 61, 64, 66, 68, 70, 72, 73), multiple vaginal examinations (7 studies) (45, 53, 61, 64, 66–68), performance of ANC (13 studies) (45, 48, 50, 55, 61, 64, 65, 67–71), prolonged rupture of membranes (18 studies) (31, 33, 45, 46, 52, 53, 56, 61, 62, 65–73), and mode of delivery (14 studies) (31, 32, 35, 44, 46, 48, 52, 61, 62, 64, 66–68, 72). The neonatal factors reported were Apgar (18 studies) (31, 33–35, 44–46, 48, 52, 56, 61, 62, 64–66, 68, 69, 71), prematurity (17 studies) (31, 33–35, 44–46, 50, 52, 53, 61, 62, 64–67, 71), meconium amniotic fluid (12 studies) (31, 45, 46, 52, 61, 64–69, 72), birth weight (17 studies) (31–35, 44–48, 52, 53, 61, 62, 64–69, 72), neonatal resuscitation (09 studies) (33–35, 52, 53, 62, 65, 66, 71), neonatal age < 7 days (11 studies) (36, 38, 48, 53, 57, 65, 66, 69, 72, 75), and neonatal sex (14 studies) (32, 34, 35, 45, 46, 48, 53, 55, 61, 62, 65, 66, 72, 73) (see Table 7).

**Table 7:** Risk factors included in the meta-analysis for neonatal sepsis.

Results	Comparison	Number studies	Effect size	Pooled estimate	Q	Heterogeneity I^**2**^, value of *p*
Apgar	<7 or >7	18	Odds ratio	2.38 (1.61–3.53)	151.21	88.8%, 0.000
Preterm	<37 or >37	17	Odds ratio	1.36 (0.81–2.26)	221.03	92.8%, 0.235
Birth weight	<2.5 kg >2.5 kg	17	Odds ratio	1.28 (0.85–1.93)	210.79	90.5%, 0.228
Mode of delivery	CS/VD	14	Odds ratio	1.05 (0.67–1.65)	93.07	86.0%, 0.807
Amios	Yes or No	12	Odds ratio	2.9 (1.83–4.58)	51.37	78.6%, 0.000
PROM	>12H, <12H	18	Odds ratio	2.8 (1.96–4.18)	99.27	82.9%, 0.000
Sex	Male/Female	14	Odds ratio	1.2 (1.1–1.42)	11.92	0.0%, 0.001
CPN	<4, ≥4	13	Odds ratio	1.4 (0.82–2.18)	67.69	82.3%, 0.170
Vaginal examination	>5, <5	7	Odds ratio	1.2 (0.51–3.09)	126.14	95.2%, 0.616
Intrapartum fever	Yes or No	13	Odds ratio	2.4 (1.52–3.75)	76.14	84.2%, 0.000
Age of newborn	<7 days, >7 days	11	Odds ratio	0.9 (0.52–1.89)	108.72	90.8%, 0.995
Newborn resuscitation	Yes, No	9	Odds ratio	1,7 (0.94–3.30)	94.67	91.5%, 0.076
Parity	Primiparous, multiparous	10	Odds ratio	1.2 (0.83–1.69)	36.48	75.3%, 0.324
History of maternal UTI	Yes, No	13	Odds ratio	2.76 (1.38–5.21)	114.73	89.5%, 0.003
Prolonged labor	>12H, <12H	5	Odds ratio	1.8 (1.47–1.90)	40.15	90.0%, 0.128

### Associated factors with maternal and neonatal sepsis

#### Factors associated with maternal sepsis

Rural residence (OR: 2.32, IC 95%: 1.01–10.9, I2 = 90.3%), parity (OR: 0.5, IC95%: 0.3–0.7, I^2^ = 0%), prolonged labor (OR: 3.4, IC95%: 1.6–6.9, I2 = 81.5%), and multiple vaginal examinations (OR: 4.4, IC95%: 1.3–14.3, I2 = 90.4%) were independently associated with maternal sepsis.

#### Factors associated with neonatal sepsis

The meta-analysis identified 6 factors as being significantly associated with neonatal sepsis, including Apgar score < 7 (OR: 2.4, CI95%: 1.6–3.5, I2 = 88.8%), the presence of meconium in the amniotic fluid (OR: 2.9, CI95%: 1.8–4.5, I2 = 78.6%), prolonged rupture of membranes >12 h (OR: 2.8, IC95%: 1.9–4.1, I2 = 82.9%), male sex (OR: 1.2, IC95%: 1.1–1.4, I2 = 0%), intrapartum fever (OR: 2.4, IC95%: 1.5–3.7, I2 = 84.2%), and history of urinary tract infection in the mother (OR: 2.76, IC95%: 1.4–5.2, I2 = 89.5%).

## Discussion

Knowledge of the burden and risk factors of maternal and neonatal sepsis is crucial for developing preventive measures and reducing maternal and neonatal mortality. This systematic review with meta-analysis filled the gaps in the literature on the prevalence of maternal and neonatal sepsis and associated factors in SSA. Based on 39 studies included in the final analysis, we found a pooled prevalence of 19.21% for maternal sepsis and 36.02% for neonatal sepsis. However, considerable heterogeneity was observed.

Factors such as rural residence, parity, prolonged labor, and multiple vaginal examinations significantly increased the risk of maternal sepsis in our study. The factors associated with neonatal sepsis in this review are classified into maternal and neonatal factors. Maternal factors such as prolonged rupture of membranes > 12 h, intrapartum fever, and history of maternal urinary tract infection and neonatal factors such as Apgar score < 7, presence of meconium in amniotic fluid, and male sex were significantly associated with neonatal sepsis. Our estimate confirms that maternal and neonatal sepsis is a major public health problem in SSA. The pooled prevalence of maternal sepsis in our review is in line with other reviews conducted in 2009 (77) and 2021 (20). This high prevalence can be explained by various factors, such as the coverage of childbirth in health facilities, the asepsis and hygiene of surfaces and materials used, the personal hygiene of pregnant women, and certain harmful practices. Our study is the first to examine both the extent and the factors associated with maternal and neonatal sepsis, which sets it apart from other reviews. It identified other factors, such as rural residence and parity, which were not identified in the 2022 review or the WHO guidelines (78). Our results could serve as a broader database for the development of interventions for the prevention and management of maternal and neonatal sepsis.

All the maternal sepsis studies included were from East Africa. The subgroup analysis of the prevalence of maternal sepsis ranged from 15.43% based on clinical signs and biology to 25.33% based on clinical signs.

The subgroup analysis of the prevalence of neonatal sepsis varied from one subdivision to another. East Africa recorded the highest prevalence, 42.96%, which was higher than the pooled prevalence. This could be explained by the weakness of the health system, the quality of services offered, and socio-cultural factors. The under-analysis of sepsis in other parts of Africa, due to the small number of studies carried out there, masks a probable high prevalence in these countries, as areas of high prevalence in these countries have probably not been covered by the small number of studies published. The prevalence of neonatal sepsis was 44.11% for the diagnostic criterion based on clinical signs and 31.25% for clinical signs and biology. Despite the various subgroup analyses, they did not make it possible to explore the source of the heterogeneity. This could be explained by the heterogeneous definition of sepsis in the different studies. The clinical signs differed from one study to another, as did the laboratory tests, which is why a meta-analysis of the individual data was necessary to identify a single algorithm.

The factors associated with maternal sepsis in our review are partly in accordance with a review of the literature in other systematic reviews in 2009 and 2022 (27, 77), where the risk factors identified were intrapartum maternal fever, multiple vaginal examinations, foul-smelling vaginal discharge, prematurity, prolonged rupture of membranes, prolonged labor, and multiple vaginal touches.

Living in a rural area was significantly associated with maternal sepsis. Women living in rural areas were 2.3 times more likely to develop sepsis than those living in urban areas. The possible explanation could be poor hygiene levels, lack of water sources, poor cord care, overcrowding in homes, and low education level of mothers in rural areas (73, 75, 79). Another possible explanation could be the fact that mothers living in urban areas are close to health facilities and have various means of transport to get to these facilities, and the availability of qualified staff and adequate technical platforms in urban areas. A vaginal examination ≥ 5 times was significantly associated with maternal sepsis. The probability of maternal sepsis was 4.4 times higher in women who had undergone a vaginal examination ≥ 5 times compared with those who had not. During vaginal examinations, there is a high likelihood of microorganisms ascending from the lower to the upper genital tract, which can lead to sepsis. Simple interventions accepted to reduce the incidence of maternal sepsis are using sterile and aseptic technical equipment by providers (hand washing, sterile drapes and instruments, and sterile gloves).

The factors associated with neonatal sepsis in our review are in line with other reviews in Pakistan (78), East Africa (21), SSA (27), and India (80), which reported maternal factors such as intrapartum maternal fever, prolonged rupture of water membranes, gestational age < 37 weeks, prolonged labor, multiple vaginal touches, history of maternal urinary tract infection and low socioeconomic status, neonatal factors such as resuscitation at birth, low birth weight < 2.5 kg, Apgar score < 7, absence of crying immediately after birth, meconium-stained amniotic fluid, and male sex.

Intrapartum fever was significantly associated with maternal sepsis. The probability of maternal sepsis was 2.4 times higher in women with a fever during pregnancy than in those without a fever. Intrapartum fever is suggestive of a maternal infection that is transmitted to the baby *in utero* or during passage through the genital tract, leading to sepsis.

Membrane rupture > 12 h significantly increased the risk of neonatal sepsis, and a history of urinary tract infection in the mother was significantly associated with neonatal sepsis. The water membrane protects the upper genital tract; when it is ruptured, bacteria can proliferate through the dilated cervix into the upper internal genital tract, causing infection. These pathogens also colonize the birth canal, which could contaminate the newborn during passage through the canal. Intrapartum antibiotic prophylaxis has been recommended as an effective practice for at-risk mothers to reduce sepsis worldwide (81).

Having an Apgar score < 7 significantly increased the risk of neonatal sepsis. As a result, the probability of neonatal sepsis was 2.4 times higher in neonates with an Apgar score < 7 compared with those with an Apgar score > 7. This finding is consistent with studies from Iraq (82) and Indonesia (83). Neonates with low Apgar scores tend to have a poor adaptation to extrauterine life due to the stress experienced during labor and therefore are more prone to infection. In addition, resuscitation procedures following birth asphyxia tend to expose newborns to pathogenic microbes. In our study, however, we did not assess birth asphyxia as a risk factor for neonatal sepsis.

Male sex has been identified as a factor associated with neonatal sepsis, and this finding has been made in the journals in India and SSA. However, we suggest further research into this factor to give a more rational explanation.

## Strengths and limitations

Our review used an appropriate search strategy, with the combination of global and regional databases reducing the risk of missing relevant regional studies. Duplicate screening and data extraction, as well as rigorous quality assessment of included data and subgroup analysis, was also performed. The relatively high number of included studies for neonatal sepsis is a strength. The number of articles included was small for maternal sepsis (7), which may limit the generalizability of the results. Most of the studies were cross-sectional, which could be a limitation. The majority of studies included in this systematic review were from East Africa, which may affect the generalizability of our results to sub-Saharan Africa. There were also publication biases between studies and a high degree of heterogeneity. We believe that this is an important subject that has not been sufficiently explored and that a meta-analysis of individual data will be necessary.

## Conclusion

In our review, the prevalence of maternal and neonatal sepsis was high. Several factors were significantly associated with this prevalence, which could help prevent maternal and neonatal sepsis by developing appropriate standard infection prevention techniques, reducing certain harmful practices, and reducing susceptibility to infection by improving maternal health through nutritional supplementation and treating infections during pregnancy. However, there is a need for evidence on other important risk factors for maternal and neonatal sepsis, including in the community. Given the high risk of bias and high heterogeneity, further high-quality research is needed in the sub-Saharan African context.

## Data availability statement

The raw data supporting the conclusions of this article will be made available by the authors, without undue reservation.

## Author contributions

FT: Conceptualization, Formal analysis, Investigation, Methodology, Software, Supervision, Writing – original draft, Writing – review & editing. CS: Conceptualization, Methodology, Writing – review & editing. ED: Conceptualization, Writing – review & editing, Investigation. BC: Investigation, Methodology, Writing – review & editing. SS: Software, Writing – review & editing, Formal analysis. ADi: Formal analysis, Validation, Writing – review & editing. ND: Conceptualization, Investigation, Writing – review & editing. BL: Methodology, Writing – review & editing. MA: Conceptualization, Writing – review & editing. KK: Formal analysis, Software, Supervision, Writing – review & editing. AT: Supervision, Writing – review & editing. AC: Writing – review & editing. ADe: Supervision, Validation, Writing – review & editing. HS: Supervision, Validation, Writing – review & editing. IT: Formal analysis, Writing – review & editing.

## References

[1] Galvão A Braga AC Gonçalves DR Guimarães JM Braga J. Sepsis during pregnancy or the postpartum period. J Obstet Gynaecol. (2016) 36:735–43. doi: 10.3109/01443615.2016.1148679 27152968

[2] Otu A Nsutebu EF Hirst JE Thompson K Walker K Yaya S. How to close the maternal and neonatal sepsis gap in sub-Saharan Africa. BMJ Glob Health. (2020) 5:e002348. doi: 10.1136/bmjgh-2020-002348, PMID: 32399259 PMC7204918

[3] Sands K Spiller OB Thomson K Portal EAR Iregbu KC Walsh TR. Early-onset neonatal Sepsis in low- and middle-income countries: current challenges and future opportunities. Infect Drug Resist. (2022) 15:933–46. doi: 10.2147/IDR.S294156, PMID: 35299860 PMC8921667

[4] Assemie MA Alene M Yismaw L Ketema DB Lamore Y Petrucka P . Prevalence of neonatal Sepsis in Ethiopia: a systematic review and Meta-analysis. Int J Pediatr. (2020) 2020:e6468492:1–9. doi: 10.1155/2020/6468492 PMC7180396 32351579

[5] Kendle AM Salemi JL Tanner JP Louis JM. Delivery-associated sepsis: trends in prevalence and mortality. Am J Obstet Gynecol. (2019) 220:391.e1–391.e16. doi: 10.1016/j.ajog.2019.02.002, PMID: 30786257

[6] Beck C Gallagher K Taylor LA Goldstein JA Mithal LB Gernand AD. Chorioamnionitis and risk for maternal and neonatal Sepsis: a systematic review and Meta-analysis. Obstet Gynecol. (2021) 137:1007–22. doi: 10.1097/AOG.0000000000004377, PMID: 33957655 PMC8905581

[7] Tesfaye T Samuel S Lera T. Determinants of puerperal sepsis among postpartum women at public hospitals of Hawassa city, southern Ethiopia: institution-based unmatched case-control study. Heliyon. (2023) 9:e14809. doi: 10.1016/j.heliyon.2023.e14809, PMID: 37025872 PMC10070673

[8] Fleischmann-Struzek C Goldfarb DM Schlattmann P Schlapbach LJ Reinhart K Kissoon N. The global burden of paediatric and neonatal sepsis: a systematic review. Lancet Respir Med. (2018) 6:223–30. doi: 10.1016/S2213-2600(18)30063-8, PMID: 29508706

[9] Reinhart K Daniels R Kissoon N Machado FR Schachter RD Finfer S. Recognizing Sepsis as a Global Health priority — a WHO resolution. N Engl J Med. (2017) 377:414–7. doi: 10.1056/NEJMp1707170, PMID: 28658587

[10] HAA AL-K Hameed RH. Sepsis during pregnancy in the postpartum duration. J Crit Rev. (2020) 7:2435–9.

[11] Bonet M Brizuela V Abalos E Cuesta C Baguiya A Chamillard M . Frequency and management of maternal infection in health facilities in 52 countries (GLOSS): a 1-week inception cohort study. Lancet Glob Health. (2020) 8:e661–71. doi: 10.1016/S2214-109X(20)30109-1, PMID: 32353314 PMC7196885

[12] Brizuela V Bonet M Romero CLT Abalos E Baguiya A Fawole B . Early evaluation of the ‘STOP SEPSIS!‘WHO global maternal Sepsis awareness campaign implemented for healthcare providers in 46 low, middle and high-income countries. BMJ Open. (2020) 10:e036338. doi: 10.1136/bmjopen-2019-036338, PMID: 32444432 PMC7247401

[13] Brizuela V Cuesta C Bartolelli G Abdosh AA Abou Malham S Assarag B . Availability of facility resources and services and infection-related maternal outcomes in the WHO global maternal Sepsis study: a cross-sectional study. Lancet Glob Health. (2021) 9:e1252–61. doi: 10.1016/S2214-109X(21)00248-5, PMID: 34273300 PMC8370881

[14] Say L Chou D Gemmill A Tunçalp Ö Moller AB Daniels J . Global causes of maternal death: a WHO systematic analysis. Lancet Glob Health. (2014) 2:e323–33. doi: 10.1016/S2214-109X(14)70227-X 25103301

[15] Ebener S Stenberg K Brun M Monet JP Ray N Sobel HL . Proposing standardised geographical indicators of physical access to emergency obstetric and newborn care in low-income and middle-income countries. BMJ Glob Health. (2019) 4:e000778. doi: 10.1136/bmjgh-2018-000778, PMID: 31354979 PMC6623986

[16] Hug L Alexander M You D Alkema L. National, regional, and global levels and trends in neonatal mortality between 1990 and 2017, with scenario-based projections to 2030: a systematic analysis. Lancet Glob Health. (2019) 7:e710–20. doi: 10.1016/S2214-109X(19)30163-9, PMID: 31097275 PMC6527519

[17] Nyenga AM. Trends in neonatal mortality in Lubumbashi (Democratic Republic of Congo) from 2011 to 2018. Pediatr Surg. (2019) 2:5. doi: 10.4236/oje.2021.115029

[18] Hall J Adams NH Bartlett L Seale AC Lamagni T Bianchi-Jassir F . Maternal disease with group B Streptococcus and serotype distribution worldwide: systematic review and Meta-analyses. Clin Infect Dis. (2017) 65:S112–24. doi: 10.1093/cid/cix660, PMID: 29117328 PMC5850000

[19] Yaya S Bishwajit G Okonofua F Uthman OA. Under five mortality patterns and associated maternal risk factors in sub-Saharan Africa: a multi-country analysis. PLoS One. (2018) 13:e0205977. doi: 10.1371/journal.pone.0205977, PMID: 30359408 PMC6201907

[20] Melkie A Dagnew E. Burden of puerperal sepsis and its associated factors in Ethiopia: a systematic review and meta-analysis. Arch Public Health. (2021) 79:216. doi: 10.1186/s13690-021-00732-y, PMID: 34844656 PMC8628469

[21] Abate BB Kasie AM Reta MA Kassaw MW. Neonatal sepsis and its associated factors in East Africa: a systematic review and meta-analysis. Int J Public Health. (2020) 65:1623–33. doi: 10.1007/s00038-020-01489-x, PMID: 32997150

[22] Kajeguka DC Mrema NR Mawazo A Malya R Mgabo MR. Factors and causes of puerperal Sepsis in Kilimanjaro, Tanzania: a descriptive study among postnatal women who attended Kilimanjaro Christian medical Centre. East Afr Health Res J. (2020) 4:158–62. doi: 10.24248/eahrj.v4i2.639, PMID: 34308233 PMC8279318

[23] Chepchirchir MV Nyamari J Keraka M. Associated factors with puerperal Sepsis among reproductive age women in Nandi County, Kenya. JMRH. (2017) 5:9348. doi: 10.22038/jmrh.2017.9348

[24] Edwards W Dore S van Schalkwyk J Armson BA. Prioritizing maternal Sepsis: National Adoption of an obstetric early warning system to prevent morbidity and mortality. J Obstet Gynaecol Can. (2020) 42:640–3. doi: 10.1016/j.jogc.2019.11.072, PMID: 32171506

[25] Cheshire J Jones L Munthali L Kamphinga C Liyaya H Phiri T . The FAST-M complex intervention for the detection and management of maternal sepsis in low-resource settings: a multi-site evaluation. BJOG Int J Obstet Gynaecol. (2021) 128:1324–33. doi: 10.1111/1471-0528.16658 33539610

[26] Shifera N Dejenie F Mesafint G Yosef T. Risk factors for neonatal sepsis among neonates in the neonatal intensive care unit at Hawassa university comprehensive specialized hospital and Adare general Hospital in Hawassa City, Ethiopia. Front Pediatr. (2023) 11:671. doi: 10.3389/fped.2023.1092671, PMID: 37138573 PMC10149989

[27] Bech CM Stensgaard CN Lund S Holm-Hansen C Brok JS Nygaard U . Risk factors for neonatal sepsis in sub-Saharan Africa: a systematic review with meta-analysis. BMJ Open. (2022) 12:e054491. doi: 10.1136/bmjopen-2021-054491, PMID: 36253895 PMC9438195

[28] Kitessa SG Teferi Bala E Makuria M Senbeta Deriba B. Determinants of puerperal sepsis at public hospitals in West Ethiopia: a case-control study. Front Womens Health. (2021) 6. doi: 10.15761/FWH.1000207

[29] Naima S. Magnitude and risk factors for puerperal Sepsis at the Pumwani maternity hospital. [Thesis]. University of Nairobi; (2017). Available at: http://erepository.uonbi.ac.ke/handle/11295/101747

[30] Joachim A Matee MI Massawe FA Lyamuya EF. Maternal and neonatal colonisation of group B streptococcus at Muhimbili National Hospital in Dar Es Salaam, Tanzania: prevalence, risk factors and antimicrobial resistance. BMC Public Health. (2009) 9:437. doi: 10.1186/1471-2458-9-437, PMID: 19948075 PMC2791767

[31] Getabelew A Aman M Fantaye E Yeheyis T. Prevalence of neonatal Sepsis and associated factors among neonates in neonatal intensive care unit at selected governmental hospitals in Shashemene town, Oromia regional State, Ethiopia, 2017. Int J Pediatr. (2018) 2018:1–7. doi: 10.1155/2018/7801272 PMC6098917 30174698

[32] Clotilde TS Motara F Laher AE. Prevalence and presentation of neonatal sepsis at a paediatric emergency department in Johannesburg, South Africa. Afr J Emerg Med. (2022) 12:362–5. doi: 10.1016/j.afjem.2022.07.013, PMID: 36032785 PMC9396294

[33] Kiwone ML Chotta NS Byamungu D Mghanga FP. Prevalence and factors associated with neonatal sepsis among hospitalized newborns at Ruvuma, southern Tanzania. South Sudan Med J. (2020) 13:86–9.

[34] Olorukooba AA Ifusemu WR Ibrahim MS Jibril MB Amadu L Lawal BB. Prevalence and factors associated with neonatal Sepsis in a tertiary hospital, north West Nigeria. Niger Med J. (2020) 61:60–6. doi: 10.4103/nmj.NMJ_31_19, PMID: 32675896 PMC7357807

[35] Adatara P Afaya A Salia SM Afaya RA Kuug AK Agbinku E . Risk factors for neonatal Sepsis: a retrospective case-control study among neonates who were delivered by caesarean section at the trauma and specialist hospital, Winneba, Ghana. Biomed Res Int. (2018) 2018:1–7. doi: 10.1155/2018/6153501, PMID: 30662911 PMC6313993

[36] Moher D Liberati A Tetzlaff J Altman DG. Preferred reporting items for systematic reviews and Meta-analyses: the PRISMA statement. Ann Intern Med. (2009) 151:264–9. doi: 10.7326/0003-4819-151-4-200908180-00135 19622511

[37] Vu-Ngoc H Elawady SS Mehyar GM Abdelhamid AH Mattar OM Halhouli O . Quality of flow diagram in systematic review and/or meta-analysis. PLoS One. (2018) 13:e0195955. doi: 10.1371/journal.pone.0195955, PMID: 29949595 PMC6021048

[38] Madrid L Seale AC Kohli-Lynch M Edmond KM Lawn JE Heath PT . Infant group B streptococcal disease incidence and serotypes worldwide: systematic review and Meta-analyses. Clin Infect Dis. (2017) 65:S160–72. doi: 10.1093/cid/cix656, PMID: 29117326 PMC5850457

[39] Munn Z Moola S Riitano D Lisy K. The development of a critical appraisal tool for use in systematic reviews addressing questions of prevalence. Int J Health Policy Manag. (2014) 3:123–8. doi: 10.15171/ijhpm.2014.71, PMID: 25197676 PMC4154549

[40] Higgins JP Thompson SG. Quantifying heterogeneity in a meta-analysis. Stat Med. (2002) 21:1539–58. doi: 10.1002/sim.1186, PMID: 12111919

[41] Higgins JPT Thompson SG Deeks JJ Altman DG. Measuring inconsistency in meta-analyses. BMJ. (2003) 327:557–60. doi: 10.1136/bmj.327.7414.557, PMID: 12958120 PMC192859

[42] Huang X Chen M Fu R He W He Y Shentu H . Efficacy of kangaroo mother care combined with neonatal phototherapy in newborns with non-pathological jaundice: a meta-analysis. Front Pediatr. (2023) 11:1098143. doi: 10.3389/fped.2023.1098143, PMID: 37082708 PMC10112003

[43] Egger M Davey Smith G Schneider M Minder C. Bias in meta-analysis detected by a simple, graphical test. BMJ. (1997) 315:629–34. doi: 10.1136/bmj.315.7109.629, PMID: 9310563 PMC2127453

[44] G/eyesus T Moges F Eshetie S Yeshitela B Abate E. Bacterial etiologic agents causing neonatal sepsis and associated risk factors in Gondar, Northwest Ethiopia. BMC Pediatr. (2017) 17:137. doi: 10.1186/s12887-017-0892-y, PMID: 28587631 PMC5461759

[45] Bekele K Bekele F Edosa D Mekonnen M Benayew M. Magnitude and associated factors of neonatal sepsis among neonates admitted to neonatal intensive care unit of northern Oromia hospitals, Ethiopia: a multicenter cross-sectional study. Ann Med Surg. (2022) 78:103782. doi: 10.1016/j.amsu.2022.103782 PMC9127159 35620038

[46] Sorsa A. Epidemiology of neonatal Sepsis and associated factors implicated: observational study at neonatal intensive care unit of Arsi university teaching and referral hospital, south East Ethiopia. Ethiop J Health Sci. (2019) 29:333–42. doi: 10.4314/ejhs.v29i3.5, PMID: 31447501 PMC6689722

[47] Akindolire AE Tongo O Dada-Adegbola H Akinyinka O. Etiology of early onset septicemia among neonates at the university college hospital, Ibadan, Nigeria. J Infect Dev Ctries. (2016) 10:1338–44. doi: 10.3855/jidc.7830, PMID: 28036314

[48] Aku FY Akweongo P Nyarko KM Mensah LG Amegan-Aho K Kumi L . Factors associated with culture proven neonatal sepsis in the ho municipality 2016. Pan Afr Med J. (2020) 36:281. doi: 10.11604/pamj.2020.36.281.20408 33088410 PMC7545969

[49] Wale A Chelkeba L Wobie Y Abebe A. Treatment outcome and associated factors of neonatal Sepsis at Mizan Tepi university teaching hospital, south West Ethiopia: a prospective observational study. Pediatric Health Med Ther. (2021) 12:467–79. doi: 10.2147/PHMT.S322069, PMID: 34539194 PMC8443800

[50] John B David M Mathias L Elizabeth N. Risk factors and practices contributing to newborn sepsis in a rural district of eastern Uganda, august 2013: a cross sectional study. BMC Res Notes. (2015) 8:339. doi: 10.1186/s13104-015-1308-4, PMID: 26254874 PMC4529696

[51] Tewabe T Mohammed S Tilahun Y Melaku B Fenta M Dagnaw T . Clinical outcome and risk factors of neonatal sepsis among neonates in Felege Hiwot referral hospital, Bahir Dar, Amhara regional State, north West Ethiopia 2016: a retrospective chart review. BMC Res Notes. (2017) 10:265. doi: 10.1186/s13104-017-2573-1, PMID: 28693597 PMC5504561

[52] Roble AK Ayehubizu LM Olad HM. Neonatal Sepsis and associated factors among neonates admitted to neonatal intensive care unit in general hospitals, eastern Ethiopia 2020. Clin Med Insights Pediatr. (2022) 16:117955652210983. doi: 10.1177/11795565221098346 PMC9134399 35645587

[53] Agnche Z Yenus Yeshita H Abdela GK. Neonatal Sepsis and its associated factors among neonates admitted to neonatal intensive care units in primary hospitals in Central Gondar zone, Northwest Ethiopia, 2019. Infect Drug Resist. (2020) 13:3957–67. doi: 10.2147/IDR.S276678, PMID: 33177846 PMC7650015

[54] Admas A Gelaw B BelayTessema WA Melese A. Proportion of bacterial isolates, their antimicrobial susceptibility profile and factors associated with puerperal sepsis among post-partum/aborted women at a referral Hospital in Bahir Dar, Northwest Ethiopia. Antimicrob Resist Infect Control. (2020) 9:14. doi: 10.1186/s13756-019-0676-2, PMID: 31956403 PMC6958633

[55] Birrie E Sisay E Tibebu NS Tefera BD Zeleke M Tefera Z. Neonatal Sepsis and associated factors among newborns in Woldia and Dessie comprehensive specialized hospitals, north-East Ethiopia, 2021. Infect Drug Resist. (2022) 15:4169–79. doi: 10.2147/IDR.S374835, PMID: 35937781 PMC9354861

[56] Yismaw AE Abebil TY Biweta MA Araya BM. Proportion of neonatal sepsis and determinant factors among neonates admitted in University of Gondar comprehensive specialized hospital neonatal intensive care unit Northwest Ethiopia 2017. BMC Res Notes. (2019) 12:542. doi: 10.1186/s13104-019-4587-3, PMID: 31455414 PMC6712769

[57] Ogunlesi TA Ogunfowora OB. Predictors of mortality in neonatal septicemia in an underresourced setting. J Natl Med Assoc. (2010) 102:915–22. doi: 10.1016/S0027-9684(15)30710-0, PMID: 21053706

[58] Ogundare E Akintayo A Aladekomo T Adeyemi L Ogunlesi T Oyelami O. Presentation and outcomes of early and late onset neonatal sepsis in a Nigerian hospital. Afr Health Sci. (2019) 19:2390–9. doi: 10.4314/ahs.v19i3.12, PMID: 32127809 PMC7040286

[59] West BA Peterside O. Sensitivity pattern among bacterial isolates in neonatal septicaemia in port Harcourt. Ann Clin Microbiol Antimicrob. (2012) 11:7. doi: 10.1186/1476-0711-11-7, PMID: 22449249 PMC3355022

[60] Atlaw D Seyoum K Handiso D Berta M. Puerperal sepsis and associated factors among women attending postnatal care service at University of Gondar Referral Hospital. Int J Pregnancy Child Birth. (2019) 5:190–5. doi: 10.15406/ipcb.2019.05.00175

[61] Alemayehu A Alemayehu M Arba A Abebe H Goa A Paulos K . Predictors of neonatal Sepsis in hospitals at Wolaita Sodo town, southern Ethiopia: institution-based unmatched case-control study, 2019. Int J Pediatr. (2020) 2020:1–10. doi: 10.1155/2020/3709672 PMC7647789 33178290

[62] Bejitual K Fikre R Ashegu T Zenebe A. Determinants of neonatal sepsis among neonates admitted to the neonatal intensive care unit of public hospitals in Hawassa City administration, Sidama region, Ethiopia, 2020: an unmatched, case-control study. BMJ Open. (2022) 12:e056669. doi: 10.1136/bmjopen-2021-056669 PMC9066491 35504644

[63] Demisse GA Sifer SD Kedir B Fekene DB Bulto GA. Determinants of puerperal sepsis among post partum women at public hospitals in west SHOA zone Oromia regional STATE, Ethiopia (institution BASEDCASE control study). BMC Pregnancy Childbirth. (2019) 19:95. doi: 10.1186/s12884-019-2230-x, PMID: 30885159 PMC6423770

[64] Teshome G Hussen R Abebe M Melaku G Wudneh A Molla W . Factors associated with early onset neonatal sepsis among neonates in public hospitals of Sidama region, southern Ethiopia, 2021: unmatched case control study. Ann Med Surg. (2022) 81:104559. doi: 10.1016/j.amsu.2022.104559 PMC9486852 36147156

[65] Adatara P Afaya A Salia SM Afaya RA Konlan KD Agyabeng-Fandoh E . Risk factors associated with neonatal Sepsis: a case study at a specialist Hospital in Ghana. Sci World J. (2019) 2019:1–8. doi: 10.1155/2019/9369051 PMC6332869 30692878

[66] Gebremedhin D Berhe H Gebrekirstos K. Risk factors for neonatal Sepsis in public hospitals of Mekelle City, North Ethiopia, 2015: unmatched case control study. PLoS One. (2016) 11:e0154798. doi: 10.1371/journal.pone.0154798, PMID: 27163290 PMC4862626

[67] Masanja PP Kibusi SM Mkhoi ML. Predictors of early onset neonatal Sepsis among neonates in Dodoma, Tanzania: a case control study. J Trop Pediatr. (2020) 66:257–66. doi: 10.1093/tropej/fmz062, PMID: 31539064

[68] Akalu TY Gebremichael B Desta KW Aynalem YA Shiferaw WS Alamneh YM. Predictors of neonatal sepsis in public referral hospitals, Northwest Ethiopia: a case control study. PLoS One. (2020) 15:e0234472. doi: 10.1371/journal.pone.0234472, PMID: 32579580 PMC7314009

[69] Dirirsa DE Dibaba Degefa B Gonfa AD. Determinants of neonatal sepsis among neonates delivered in Southwest Ethiopia 2018: a case-control study. SAGE Open Med. (2021) 9:205031212110270. doi: 10.1177/20503121211027044 PMC8237212 34249361

[70] Kayom VO Mugalu J Kakuru A Kiguli S Karamagi C. Burden and factors associated with clinical neonatal sepsis in urban Uganda: a community cohort study. BMC Pediatr. (2018) 18:355. doi: 10.1186/s12887-018-1323-4, PMID: 30424740 PMC6234629

[71] Alemu M Ayana M Abiy H Minuye B Alebachew W Endalamaw A. Determinants of neonatal sepsis among neonates in the northwest part of Ethiopia: case-control study. Ital J Pediatr. (2019) 45:150. doi: 10.1186/s13052-019-0739-2, PMID: 31779698 PMC6883598

[72] Kayange N Kamugisha E Mwizamholya DL Jeremiah S Mshana SE. Predictors of positive blood culture and deaths among neonates with suspected neonatal sepsis in a tertiary hospital, Mwanza-Tanzania. BMC Pediatr. (2010) 10:39. doi: 10.1186/1471-2431-10-39, PMID: 20525358 PMC2889942

[73] Ogunlesi TA Ogunfowora OB Osinupebi O Olanrewaju DM. Changing trends in newborn sepsis in Sagamu, Nigeria: bacterial aetiology, risk factors and antibiotic susceptibility. J Paediatr Child Health. (2011) 47:5–11. doi: 10.1111/j.1440-1754.2010.01882.x, PMID: 20973858

[74] Ogundare E Akintayo A Florence D Okeniyi J Adeyemi A Ogunlesi T . Neonatal Septicaemia in a rural Nigerian hospital: Aetiology, presentation and antibiotic sensitivity pattern. Br J Med Med Res. (2016) 12:1–11. doi: 10.9734/BJMMR/2016/22325

[75] Siakwa M Kpikpitse D Mupepi S Semuatu M. Neonatal Sepsis in rural Ghana: a case control study of risk factors in a birth cohort. Peer Reviewed Articles. (2014); Available at: https://scholarworks.gvsu.edu/kcon_articles/43

[76] Sterne JAC Sutton AJ Ioannidis JPA Terrin N Jones DR Lau J . Recommendations for examining and interpreting funnel plot asymmetry in meta-analyses of randomised controlled trials. BMJ. (2011) 343:d4002. doi: 10.1136/bmj.d4002, PMID: 21784880

[77] Seale AC Mwaniki M Newton CRJC Berkley JA. Maternal and early onset neonatal bacterial sepsis: burden and strategies for prevention in sub-Saharan Africa. Lancet Infect Dis. (2009) 9:428–38. doi: 10.1016/S1473-3099(09)70172-0, PMID: 19555902 PMC2856817

[78] Alam MM Saleem AF Shaikh AS Munir O Qadir M. Neonatal sepsis following prolonged rupture of membranes in a tertiary care hospital in Karachi, Pakistan. J Infect Dev Ctries. (2014) 8:067–73. doi: 10.3855/jidc.3136 24423714

[79] Onyedibe KI Utoh-Nedosa AU Okolo M Onyedibe KIO Ita OI Udoh UA . Impact of socioeconomic factors on neonatal Sepsis in Jos. Nigeria Jos J Med. (2012) 6:54–8.

[80] Murthy S Godinho MA Guddattu V Lewis LES Nair NS. Risk factors of neonatal sepsis in India: a systematic review and meta-analysis. PLoS One. (2019) 14:e0215683. doi: 10.1371/journal.pone.0215683, PMID: 31022223 PMC6483350

[81] Tewari V. Current evidence on prevention and Management of Early Onset Neonatal Sepsis. J Infect Dis Ther. (2016) 4:2–5.

[82] Mohamed DA Ibrahim SA Suleman SK. Risk factors associated with early and late-onset of neonatal Sepsis in Duhok City. Erbil J Nurs Midwifery. (2020) 3:1–10. doi: 10.15218/ejnm.2020.01

[83] Hayun M Alasiry E Daud D Febriani DB Madjid D. The risk factors of early onset neonatal sepsis. Am J Clin Exp Med. (2015) 3:78–82. doi: 10.11648/j.ajcem.20150303.11

